# Translation and Cultural Adaptation into Arabic of Patient-Reported Outcome Measurement Information System^®^ Item Banks: Cognitive Function Abilities and Physical Function for Samples with Mobility Aid Users

**DOI:** 10.3390/healthcare12020211

**Published:** 2024-01-15

**Authors:** Hadeil S. Almohaya, Hadeel R. Bakhsh, Bodor Bin Sheeha, Monira I. Aldhahi, Rehab Alhasani

**Affiliations:** Department of Rehabilitation Sciences, College of Health and Rehabilitation Sciences, Princess Nourah bint Abdulrahman University (PNU), P.O. Box 84428, Riyadh 11671, Saudi Arabiahrbakhsh@pnu.edu.sa (H.R.B.); bhbinsheeha@pnu.edu.sa (B.B.S.); mialdhahi@pnu.edu.sa (M.I.A.)

**Keywords:** patient-reported outcomes, PROMIS, cognitive function, cognitive abilities, mobility, physical function, user aids

## Abstract

Purpose: This study aimed to provide Arabic-speaking individuals with tools to assess their cognitive abilities and physical function and to contribute to a better understanding of these capabilities in this population. Thus, the specific objective was to translate into Arabic and culturally adapt two Patient-Reported Outcome Measurement Information System (PROMIS) item banks: the Adult Cognitive Function Abilities and the Physical Function for Samples with Mobility Aid Users item banks. This study employed the Functional Assessment of Chronic Illness Therapy (FACIT) multilingual translation methodology to ensure cultural and linguistic relevance. The translation process included forward and back translations, expert reviews, and finalisation by a language coordinator. Cognitive debriefing interviews were conducted with 30 native healthy Arabic speakers to assess the clarity and comprehension of translated items. Most items were well understood, but two items related to cognitive ability and four related to physical functions required revision to address participant confusion. The translations were refined based on the participants’ feedback and expert recommendations. This study followed a rigorous translation process and included cognitive debriefing interviews to ensure linguistic and cultural equivalence. The availability of these tools in Arabic enhances cross-cultural research and practice in healthcare and contributes to a global understanding of cognitive and physical functions.

## 1. Introduction

Cognitive and physical function play critical roles in enabling individuals to undertake everyday tasks and maintain their overall well-being [1,2] and quality of life [3,4]. Cognitive function encompasses a wide range of mental processes, including attention, memory, executive functions, and information processing [5], whereas physical function refers to the capacity to engage in both simple and instrumental activities of daily living [6]. Cognitive function is linked to physical function as cognitive abilities are closely tied to one’s ability to perform daily activities that require physical coordination and mobility [7]. In particular, individuals who rely on mobility aids face unique challenges in maintaining physical function, and their cognitive abilities play a crucial role in successful adaptation [8].

Given the vital roles of cognitive and physical functions, assessments of these functions are essential for understanding an individual’s abilities, identifying impairments, and monitoring treatment outcomes in various populations [8,9]. In addition to objective measures, subjective measures that capture individuals’ perceptions of their cognitive and physical abilities can provide valuable insights into their functional abilities, overall quality of life, and well-being [10,11]. Patient-Reported Outcome Measures (PROMs) have gained increasing recognition as important tools for assessing subjective experiences in healthcare research and practice. The Patient-Reported Outcome Measurement Information System (PROMIS) initiative developed by the National Institutes of Health (NIH) has created a comprehensive set of item banks to assess various health domains. These item banks consist of a calibrated pool of questions designed to measure specific constructs, providing efficient and precise assessments of patients’ experiences [12].

For example, the PROMIS Cognitive Function Abilities and Physical Function for Samples with Mobility Aid Users item banks were designed to capture self-reported cognitive and physical abilities across populations and cultural contexts, respectively. The psychometric properties of these measures, including reliability and validity, have been demonstrated in diverse populations [13,14,15,16,17,18]. However, for the successful implementation of PROMIS across cultural and linguistic contexts, it is crucial to not only translate the measures accurately but also to adapt them to the culture of the target population [19].

PROMIS measures, spanning over 60 languages and increasingly employed globally [20], have witnessed successful translation and application in diverse populations. It is noteworthy that the Dutch–Flemish adaptation encompasses more than 30 domains and has been validated across various demographic groups [20]. Although the integration of PROMIS outcome measures in an Arabic clinical practice is advancing, it remains a rare occurrence. Several studies spanning different populations have underscored the necessity of considering linguistic and cultural intricacies during the translation process to ensure conceptual equivalence [21,22,23,24].

Despite the expanding recognition of PROMIS, a research gap persists in the focused exploration of translating and adapting PROMIS measures for cognitive and physical functions within the Arabic-speaking community. Recognising the profound impact of language and cultural factors on individuals’ health perceptions [25], there is imperative to forge culturally relevant and linguistically precise measures for Arabic-speaking individuals. This step is vital to uphold the validity and applicability of PROMIS within this specific population, aligning with the diverse conceptions of well-being, such as harmony, social connections, and the balance of mind and body prevalent in the Middle East [26].

Thus, this study aimed to translate and culturally adapt the PROMIS Adult Cognitive Function Abilities and Physical Function for Samples with Mobility Aid Users item banks into Arabic. By doing so, we aimed to provide Arabic-speaking individuals with tools to gain insights into their cognitive and physical functioning that can facilitate appropriate interventions and support. Moreover, the availability of standardised measures in Arabic will enable cross-cultural comparisons and the pooling of data from diverse populations, enhancing our understanding of cognitive and physical function in a global context.

## 2. Methods

Cross-cultural translation and cultural adaptation processes were used to translate the PROMIS Adult Cognitive Function Abilities and Physical Function for Samples with Mobility Aid Users item banks into the Arabic language. 

### 2.1. Measures 

#### 2.1.1. PROMIS Cognitive Function Abilities Item Bank

The item bank for PROMIS Cognitive Function Abilities (version 2.0) comprises 31 items designed to assess an individual’s cognitive abilities in completing tasks related to memory, thinking, concentration, and language skills over the past seven days. The response options ranged from ‘not at all [able]’ to ‘very much [able]’. Higher scores indicate better perceived cognitive functioning [27].

#### 2.1.2. PROMIS Physical Function with Mobility Aids Item Bank 

The item bank for PROMIS Physical Function with Mobility (version 1.0) was created to assess an individual’s self-reported capacity to perform various physical tasks. These items are suitable for use in healthy adults and those with clinical conditions. Physical tasks encompass a broad range of activities, from instrumental activities of daily living (IADL) to more complex tasks that involve a combination of skills, often occurring in social settings. The item bank addresses different aspects, including the functioning of the upper and lower extremities as well as central regions, such as the neck and back. The response options range from (1) ‘cannot do it at all’ to (5) ‘without any difficulty’ [28].

### 2.2. Formation of the Translation Team

At the outset of the translation process, a team of skilled professionals was assembled in adherence to the specifications outlined by the PROMIS Health Organization. All members of the Arabic translation team were contracted by the Functional Assessment of Chronic Illness Therapy Translation (FACITtrans) group as linguists, translators, proofreaders, and cognitive interviewers, specifically targeting the items from the FACIT Measurement System. All team members were native speakers of Arabic, except for the backtranslator, who was a native speaker of English and fluent in Arabic. Each member of the FACITtrans Arabic translation team met the International Standard specifies (ISO) 17100 [29] for professional competencies and translation qualifications of linguists (Table 1).

### 2.3. Preparation of the Item History Template

The item history template serves as a comprehensive record of the entire translation process of the original English items. The PROMIS Health Organization guidelines emphasise the accurate and detailed documentation of each step in the FACIT translation process, along with associated comments, within the item history template. The template can be created in an Excel or Word format with specific requirements for each. In the Excel format, each item occupies its own column, whereas in the Word format, a separate page is allocated to each item. These formats facilitate vertical comparisons of specific items at different stages of translation, aiding translators and reviewers in their assessments. 

### 2.4. Translation

The widely utilised FACIT Multilingual Translation Methodology [9,30] was adopted to ensure the cultural and linguistic relevance of the Arabic versions of the item banks. The objective of this approach is to achieve cross-cultural equivalence across five domains.

1. Linguistic/semantic: This ensures that items have the same meaning in the source and translated versions.
2. Content: Ensuring the relevance of the items in both cultures.
3. Conceptual: Ensuring that the translated items measure the same theoretical constructs as the source items.
4. Criterion: Comparing the translated items to a standardised or well-known measurement to assess the similarity of measurement properties.
5. Technical: This ensures that the assessment method yields comparable measurements in both cultures.

In this study, we focused on addressing the first three dimensions. Further psychometric validation is needed to address these two dimensions. The translation team followed a systematic process to create accurate and culturally sensitive translations from English sources. The following steps were undertaken based on FACIT guidelines.

1. Two independent professional translators, both native Arabic speakers, performed forward translations of the source items from English into Arabic.
2. A third independent translator, also a native Arabic speaker, reconciled the two forward translations by creating a hybrid version. Additionally, the translator noted why they believed that the reconciled translation provided the most suitable representation of the intended meaning of the source items.
3. Without seeing the original items, a native English-speaking translator fluent in Arabic backtranslated the Arabic translation into English.
4. A translation project manager compared the back-translated English version with the original source items to identify any differences and clarify the intended meaning of the items to the reviewers. This step also allowed for an initial assessment of the harmonisation between languages.
5. Three experts, native Arabic speakers with expertise in linguistics and/or healthcare, independently reviewed the translations generated in the previous steps. The reviewers assessed the appropriateness of each translation and proposed alternate translations if the previous versions were deemed unsatisfactory.
6. The translation project manager evaluated feedback from the expert reviewers, addressed any potential issues or problems in the proposed translations, and formulated comments and questions to provide guidance to the language coordinator.
7. The language coordinator, a native speaker of Arabic, reviewed all information in the item history, including translations, backtranslations, and comments from the translation project manager and expert reviewers. Based on this evaluation, they provided the final translation as well as the literal backtranslation and polished backtranslation for each item. The language coordinator justified the final translation by explaining any deviations from the reviewers’ recommendations or reconciled version.
8. A preliminary assessment of the equivalence and accuracy of the final translation was completed by the translation project manager by comparing the original source items with the final backtranslations. In addition, the authors verified that the translation process was fully documented. Furthermore, the PROMIS Statistical Centre completed a quality review to assess consistency between the items and prior translations. The language coordinator was consulted when necessary for further input.
9. Two proofreaders independently undertook formatting, typesetting, and proofreading and reconciled any discrepancies apparent in their proofreading comments.
10. The Arabic items were tested with native Arabic speakers who participated in cognitive debriefing interviews (described in detail in the next section) to verify that the meaning of the Arabic items was equivalent to the original items.
11. The comments of the participants, which were backtranslated into English, were compiled and summarised by the translation project manager. The issues raised during cognitive testing were reviewed and solutions were proposed by the language coordinator. The translation project manager ensured that the proposed solutions harmonised with the source items.

### 2.5. Cognitive Interviews

Cognitive interviewing is a crucial step in the development and translation of measurement tools and is often overlooked by researchers. It involves examining how participants think and form answers to each question and provides insights into their understanding and interpretation of the items [31]. The main goal of cognitive interviewing is to gain an understanding of how individuals comprehend and explain items, thereby providing insights into issues that could occur when the measure is used in investigations.

For the cognitive interview process, it is important that the participants are recruited from the population for which the questionnaire was intended [32]. For the PROMIS Adult Cognitive Function Abilities and Physical Function for Samples with Mobility Aid Users item banks, the PROMIS Health Organization specifies that centring and calibration samples should come from the general population. In the current study, the pre-testing phase of the item bank was conducted with 30 healthy individuals, representing different demographics as closely as possible. The Development and Validation Scientific Standards specify interviewing at least five participants for each item [33].

During cognitive interviews conducted individually, the interviewer utilised probing techniques to facilitate discussion. Each participant assessed the items in terms of difficulty and comprehension, provided feedback on their understanding of the items, rated the level of difficulty they encountered, and explained the reasons behind their chosen answers for each item. Furthermore, participants identified confusing words and expressed them in their own words. 

This qualitative aspect of the interview provided a valuable context for recognising potential issues with item wording and cultural relevance that might affect the scale’s validity. The cognitive interviewer underwent specialised training in cognitive interviewing skills (e.g., precautions of cognitive interviewing, implementation steps, fundamental concepts), following the recommendations of the PROMIS Health Organization, and they also acted as note-takers and facilitators [34]. 

Cognitive interviews were conducted after the participants independently responded to items from the item banks. Using the interview outline, they completed the interview, which took no more than 45 min for any given participant. 

Following the interviews, participants’ suggestions and perspectives were compiled into a cognitive debriefing report. The translation project manager assessed the feedback for individual items and examined the participants’ comprehension and outlined any problems. Suggestions for revisions were discussed with the language coordinator, and final suggestions for changes or translation solutions were provided. The cognitive debriefing report was then translated into English and sent for a final quality check to the PROMIS Statistical Centre.

## 3. Results

Thirty native Arabic speakers aged between 20 and 56 years (*M* = 35.73, 50% male) participated in cognitive interviews. The participants were from five Arabic-speaking countries: Egypt (*n* = 6), Jordan (*n* = 6), Kuwait (*n* = 6), Morocco (*n* = 6), and Saudi Arabia (*n* = 6). Regarding the highest level of education, eleven participants (36.67%) had completed high school, seven (23.33%) had completed some college degree, and twelve (40%) had completed a bachelor’s degree. 

For each item, cognitive interviews were conducted with six participants. While most items from the Adult Cognitive Function Abilities item bank were well understood by the participants, two items caused confusion, leading to modifications by the translation team (Table 2). Similarly, most items from the Physical Function for Samples with Mobility Aid Users item bank were well understood; however, four items caused some confusion and were modified by the translation team (Table 3). 

## 4. Discussion

The primary objective of this study was to translate and culturally adapt the PROMIS Adult Cognitive Function Abilities and Physical Function for Samples with Mobility Aid Users item banks into Arabic to provide Arabic-speaking individuals with valid and reliable tools to assess their cognitive abilities and physical function. The process of translation and cross-cultural adaptation is a rigorous method to ensure that the Arabic version maintains linguistic and cultural equivalence. The inclusion of bilingual Arabic linguists and healthcare experts was pivotal in ensuring cultural sensitivity, methodological robustness, and upholding the intended meaning of the content for the target audience. The involvement of healthcare experts contributed to domain-specific knowledge, verifying that the translated items maintained clinical relevance and accurately captured the intended construct within the cultural context. The linguistically Arabic version of the questionnaires can be obtained through the HealthMeasures website (www.healthmeasures.net).

Cognitive interviews played a central role in confirming the comprehensibility and relevance of the questionnaire to participants, thereby further validating the adapted tool. The study’s outcomes indicate that the Arabic versions of the Cognitive Function Abilities and the Physical Function for Samples with Mobility Aid Users item banks are well suited for Arabic-speaking populations. Only a small number of test items were not well understood by the participants, which is typically related to cultural nuances (e.g., use of cutlery and food storage) or lack of specificity (e.g., using ‘lists’ to refer to to-do lists). Most items showed substantial agreement between the translators and reviewers. The participants’ strong grasp and clear comprehension underscored the success of the translation and adaptation efforts, thus accomplishing the study’s aim of enhancing their understanding of these functions among Arabic-speaking populations and facilitating appropriate interventions and support. Additionally, the availability of standardised measures in Arabic will enable cross-cultural comparisons and pooling of data from diverse populations, contributing to a global understanding of cognitive and physical functions.

One of the strengths of this study lies in the utilisation of the FACIT Multilingual Translation Methodology [27]. This widely recognised translation method ensures the cultural and linguistic relevance of the translated Arabic versions of the item banks. By following the FACIT guidelines, this study achieved cross-cultural equivalence in three dimensions: linguistic/semantic, content, and conceptual. The rigorous translation process ensured that the items maintained their intended meaning and accurately captured the cognitive and physical function constructs across languages. This study placed significant emphasis on ensuring cultural sensitivity during the translation process. By involving native Arabic speakers and experts with linguistic and healthcare backgrounds, the translations were more likely to accurately capture cultural nuances and idiomatic expressions relevant to Arabic-speaking individuals. Additionally, the translation process was systematic and comprehensive and included multiple stages of translation, reconciliation, backtranslation, expert reviews, and cognitive testing. This multistep approach allowed for rigorous scrutiny and refinement of the translated items, ensuring their accuracy, clarity, and cultural appropriateness. 

Furthermore, the inclusion of cognitive debriefing interviews and pre-testing of the scales with native Arabic speakers provided valuable insights into the participants’ understanding and interpretation of the translated items. This feedback helped identify potential areas of confusion or ambiguity, allowing for further revisions to enhance the clarity and comprehension of the Arabic versions of the items. Moreover, incorporating participants from diverse educational backgrounds presents an opportunity to explore the impact of education on the interpretation of and responses to the measures. However, it also raises concerns regarding the generalisability of the findings as varying educational levels might influence the comprehension of the items, potentially affecting response patterns and the instrument’s performance across different educational levels. Inclusion of individuals with cognitive or physical impairments can significantly enrich our understanding of an instrument’s performance. However, this may simultaneously introduce complex interpretations. For instance, individuals with cognitive impairments might encounter challenges in comprehending and responding consistently to questionnaire items, potentially affecting the validity of the scale for this subgroup. Similarly, individuals from specific clinical populations may have unique experiences or perspectives that influence their interpretation of an instrument.

Another strength of this study is the use of the PROMIS Adult Cognitive Function Abilities and Physical Function for Samples with Mobility Aid Users item banks, which have established psychometric properties [13,14,15,16,17,18]. Previous research has demonstrated the reliability and validity of item banks in diverse populations [13,14,15,16,17,18]. By utilising these well-developed items, this study ensures that the translated Arabic measure has strong psychometric properties and can effectively assess cognitive and physical abilities in Arabic-speaking populations. This strengthens the comparability of data across different cultural and linguistic contexts, promoting standardised assessments of cognitive and physical functions.

Despite the strengths of this study, it has some limitations that should be acknowledged. First, it focused on linguistic and cultural equivalence and did not address the remaining dimensions of cross-cultural equivalence, including criterion and technical equivalence. Therefore, further psychometric validation is necessary to establish the reliability and validity of the translated Arabic items. Hence, the authors are currently undertaking a psychometric methodology study using Rasch analysis to assess the validity and reliability of the Cognitive Function Abilities item bank. The Rasch analysis is a robust statistical methodology. Through item fit assessment, Rasch analysis identifies discrepancies between observed responses and the model’s expectations, discerns item difficulty or severity hierarchy, and appraises the instrument’s structural validity [35]. Additionally, it uncovers differential item functioning across diverse subgroups, ensuring measurement equivalence. Assessing these dimensions would provide a comprehensive evaluation of the psychometric properties of the Arabic versions of both scales.

Furthermore, while the inclusion of participants with diverse educational backgrounds enhanced the generalisability of our findings, we acknowledge that the scope of the study did not specifically target individuals with cognitive or physical impairments or participants with specific clinical conditions. Therefore, to ensure the applicability of the translated items, future research should investigate the psychometric properties of a diverse sample of participants with clinical conditions, such as mental disorders and neurological conditions. This expanded research approach will involve comprehensive psychometric analyses to validate the translated items and to ensure their validity and clinical utility.

In conclusion, the translation into Arabic and cultural adaptation of the PROMIS Adult Cognitive Function Abilities and Physical Function for Samples with Mobility Aid Users item banks represent an important step in providing Arabic-speaking individuals with a standardised measure to assess their cognitive and physical functioning. This study demonstrated the strengths of the FACIT translation method in achieving cross-cultural equivalence and maintaining linguistic and cultural relevance. Utilisation of these well-established PROMIS item banks further strengthens the validity and comparability of the Arabic version. However, further psychometric validation and larger-scale studies are necessary to establish measurement properties and enhance the generalisability of the findings. The availability of a valid and reliable tool in Arabic contributes to the global understanding of cognitive and physical functions and facilitates cross-cultural research and practice in healthcare.

## Figures and Tables

**Table 1:** FACITtrans Arabic translation team.

Role *	Education	Title and Profession
Translation Account Manager FACITtrans	MBA	Director, PROMIS^®^ Lead
Translation Project Coordinator FACITtrans	BA	Senior COA Translations Manager—Life Sciences, PROMIS^®^ Specialist
Translation Project Manager FACITtrans	BA, Spanish Linguistics	Senior COA Translation Project Manager—Life Sciences
Forward 1	BA, Languages and Translation, Simultaneous Interpretation (English < > Arabic)	Senior Translator, Copywriter and Proofreader Professional and Translator Interpreter
Forward 2	MA, Linguistics	Professional Translator and Interpreter
Reconciler/Proofreader	Ph.D., Linguistics	Professional Linguist and Translator
Backtranslator	MA, Diplomacy BA, Medical Technology	Professional Translator 16 years full immersion in Arabic public school system and 3.5 years of undergraduate education (nursing) at King Abdul Aziz University, Jeddah, Saudi Arabia
Reviewer 1	Ph.D., Linguistics	Linguist and researcher Pragmatics, sociolinguistics, discourse analysis, ideology, identity and translation studies
Reviewer 2	Ph.D., Linguistics	Professional Linguist and Translator
Reviewer 3, Language Coordinator, Proofreader 1	DDS MA, Biblical Studies	Professional Translator and Interpreter Close to 30 years’ experience specializing in medical, legal, and religious translation

* All members of the FACITtrans Arabic translation team are native speakers of Arabic, with the exception of the Backtranslator, who is a native English speaker and fluent in Arabic.

**Table 2:** Adjustments to items during the process of translating the Adult Cognitive Function Abilities item bank.

Source Item	Arabic Version; English Equivalent	Final Arabic Version	Reasons for Adaptation
PC43_2r	كان ذهني متوقّداً كالمعتاد; My mind has been as sharp as usual	لقد كان ذهني حادّاً كالمعتاد	One participant found the Arabic word for “sharp” unclear in the test version, so an alternative was used in the final version.
PC-CaPS19r	لقد كانت قدرتي على الإحاطة بالقوائم جيدة كالمعتاد: My ability to keep track of lists has been as good as usual	لقد كانت قدرتي على متابعة ما هو مكتوب على قائمة مهام ما جيدةً كالمعتاد	Three participants associated the test item with menus or note-taking, so the final version clarified that the focus was on to-do lists.

**Table 3:** Adjustments to items during the process of translating the Physical Function for Samples with Mobility Aid users item bank.

Source Item	Arabic Version; English Equivalent	Final Arabic Version	Reasons for Adaptation
PF_23	هل بمقدورك الوصول إلى جسمٍ مرتفع( مثل علبة حساء) وإنزاله من فوق رأسك؟; Are you able to reach and get down an object (such as a can of soup) from above your head?	هل بمقدورك الوصول إلى جسمٍ مرتفع أعلى من مستوى رأسك (مثل علبة حمّص) وإنزاله؟	Two participants reported that this item was unclear. This may be because soup is not commonly canned in Arabic-speaking countries. Therefore, garbanzo beans are instead referred to in the final version.
PF_24	هل بمقدورك حمل كيس فيه بقالة لمسافة قصيرة؟; Are you able to carry a bag of groceries for a short distance?	هل بمقدورك حمل كيس ملآن من البقالة لمسافة قصيرة؟	One participant thought that the test item referred to an empty grocery bag. Therefore, the final version refers to a bag full of groceries.
PF_33	هل بمقدورك الوصول إلى ما فوق رأسك؟; Are you able to reach above your head?	هل بمقدورك الوصول إلى جسم مرتفع أعلى من رأسك؟	Two participants felt the test item was unclear, potentially because it is uncommon in Arabic to refer to reaching without reference to an object. Therefore, the final version refers to reaching for an “object”.
PF_63	كم تواجه من صعوبة حاليًّا في وضع الأطعمة القابلة للدَّهن على الخبز باستخدام سكّين؟; How much difficulty do you currently have applying spreads to breads using a knife?	كم تواجه من صعوبة حاليًّا في وضع الأطعمة القابلة للدَّهن على الخبز باستخدام سكّين أو ملعقة؟	Two participants highlighted that they often use a spoon rather than a knife to apply spreads to bread. Therefore, the final version refers to a spoon as well as a knife.

## Data Availability

The datasets analysed during the current study are available from the corresponding author upon reasonable request.

## Abbreviations

FACIT	Functional Assessment of Chronic Illness Therapy
LC	Language coordinator
LSP	Language Service Provider
MSS	Department of Medical Social Sciences
PROMs	Patient-Reported Outcome Measures
PROMIS	Patient-Reported Outcome Measurement Information System
NIH	National Institutes of Health

## References

[1] Hung S.-T., Cheng Y.-C., Wu C.-C., Su C.-H. (2023). Examining Physical Wellness as the Fundamental Element for Achieving Holistic Well-Being in Older Persons: Review of Literature and Practical Application in Daily Life. J. Multidiscip. Healthc..

[2] Llewellyn D.J., Lang I.A., Langa K.M., Huppert F.A. (2008). Cognitive function and psychological well-being: Findings from a population-based cohort. Age Ageing.

[3] Song R., Fan X., Seo J. (2023). Physical and cognitive function to explain the quality of life among older adults with cognitive impairment: Exploring cognitive function as a mediator. BMC Psychol..

[4] Sunde S., Hesseberg K., Skelton D.A., Ranhoff A.H., Pripp A.H., Aarønæs M., Brovold T. (2021). Associations between health-related quality of life and physical function in older adults with or at risk of mobility disability after discharge from the hospital. Eur. Geriatr. Med..

[5] Kiely K.M., Michalos A.C. (2014). Cognitive Function. Encyclopedia of Quality of Life and Well-Being Research.

[6] Garber C.E., Greaney M.L., Riebe D., Nigg C.R., Burbank P.A., Clark P.G. (2010). Physical and mental health-related correlates of physical function in community dwelling older adults: A cross sectional study. BMC Geriatr..

[7] Dunsky A. (2019). The Effect of Balance and Coordination Exercises on Quality of Life in Older Adults: A Mini-Review [Mini Review]. Front. Aging Neurosci..

[8] Hunter S.W., Divine A., Omana H., Madou E., Holmes J. (2020). Development, reliability and validity of the Safe Use of Mobility Aids Checklist (SUMAC) for 4-wheeled walker use in people living with dementia. BMC Geriatr..

[9] Epstein J., Santo R.M., Guillemin F. (2015). A review of guidelines for cross-cultural adaptation of questionnaires could not bring out a consensus. J. Clin. Epidemiol..

[10] Savard J., Ganz P.A. (2016). Subjective or Objective Measures of Cognitive Functioning—What’s More Important?. JAMA Oncol..

[11] Munguía-Izquierdo D., Pulido-Martos M., Acosta F.M., Acosta-Manzano P., Gavilán-Carrera B., Rodriguez-Ayllon M., Geenen R., Delgado-Fernández M., Álvarez-Gallardo I.C., Segura-Jiménez V. (2021). Objective and subjective measures of physical functioning in women with fibromyalgia: What type of measure is associated most clearly with subjective well-being?. Disabil. Rehabil..

[12] Cella D., Yount S., Rothrock N., Gershon R., Cook K., Reeve B., Ader D., Fries J.F., Bruce B., Rose M. (2007). The Patient-Reported Outcomes Measurement Information System (PROMIS): Progress of an NIH Roadmap Cooperative Group During its First Two Years. Med. Care.

[13] Amtmann D., Bamer A., Cook K., Harniss M., Johnson K. (2010). Adapting PROMIS physical function items for users of assistive technology. Disabil. Health J..

[14] Becker H., Stuifbergen A., Lee H., Kullberg V. (2014). Reliability and Validity of PROMIS Cognitive Abilities and Cognitive Concerns Scales Among People with Multiple Sclerosis. Int. J. MS Care.

[15] Haan E.-J.A., Terwee C.B., Van Wier M.F., Willigenburg N.W., Van Deurzen DF P., Pisters M.F., Kaat A.J., Roorda L.D. (2020). Translation, cross-cultural and construct validity of the Dutch–Flemish PROMIS^®^ upper extremity item bank v2.0. Qual. Life Res..

[16] Iverson G.L., Marsh J.M., Connors E.J., Terry D.P. (2021). Normative reference values, reliability, and item-level symptom endorsement for the PROMIS^®^ v2. 0 cognitive function-short forms 4a, 6a and 8a. Arch. Clin. Neuropsychol..

[17] Oude Voshaar M.A.H., ten Klooster P.M., Taal E., Krishnan E., van de Laar M.A.F.J. (2012). Dutch translation and cross-cultural adaptation of the PROMIS^®^ physical function item bank and cognitive pre-test in Dutch arthritis patients. Arthritis Res. Ther..

[18] Valentine T.R., Weiss D.M., Jones J.A., Andersen B.L. (2019). Construct validity of PROMIS^®^ Cognitive Function in cancer patients and noncancer controls. Health Psychol..

[19] Devine J., Kaman A., Seum T.L., Zoellner F., Dabs M., Ottova-Jordan V., Schlepper L.K., Haller A.C., Topf S., Boecker M. (2023). German translation of the PROMIS^®^ pediatric anxiety, anger, depressive symptoms, fatigue, pain interference and peer relationships item banks. J. Patient-Rep. Outcomes.

[20] Terwee C.B., Roorda L.D. (2023). Country-specific reference values for PROMIS^®^ pain, physical function and participation measures compared to US reference values. Ann. Med..

[21] Schnohr C.W., Rasmussen C.L., Langberg H., Bjørner J.B. (2017). Danish translation of a physical function item bank from the Patient-Reported Outcome Measurement Information System (PROMIS). Pilot Feasibility Stud..

[22] Gao W., Yuan C. (2021). Translation and cultural adaptation of the Pediatric Patient-Reported Outcome Measurement Information System-Emotional Distress item banks into Chinese. J. Spec. Pediatr. Nurs..

[23] Chan S.W.W., Chien C.W., Wong A.Y.L., Pang M.Y.C. (2021). Translation and psychometric validation of the traditional Chinese version of patient-reported outcomes measurement information system Pediatric-25 Profile version 2.0 (PROMIS-25) in Chinese Children with Cancer in Hong Kong. Qual. Life Res..

[24] Terwee C.B., Roorda L.D., de Vet HC W., Dekker J., Westhovens R., van Leeuwen J., Cella D., Correia H., Arnold B., Perez B. (2014). Dutch–Flemish translation of 17 item banks from the Patient-Reported Outcomes Measurement Information System (PROMIS). Qual. Life Res..

[25] Mahmoud G.A., Rady H.M., Mostafa A.M. (2019). Cross cultural adaptation and validation of an Arabic version of selected PROMIS measures for use in rheumatoid arthritis patients. Egypt. Rheumatol..

[26] Joshanloo M. (2014). Eastern Conceptualizations of Happiness: Fundamental Differences with Western Views. J. Happiness Stud..

[27] Chen Y.T., Lescoat A., Khanna D., Murphy S.L. (2023). Perceived Cognitive Function in People With Systemic Sclerosis: Associations With Symptoms and Daily Life Functioning. Arthritis Care Res..

[28] Jensen R.E., Potosky A.L., Reeve B.B., Hahn E., Cella D., Fries J., Smith A.W., Keegan T.H., Wu X.-C., Paddock L. (2015). Validation of the PROMIS physical function measures in a diverse US population-based cohort of cancer patients. Qual. Life Res..

[29] (2015). Translation Services—Requirements for Translation Services.

[30] Eremenco S.L., Cella D., Arnold B.J. (2005). A Comprehensive Method for the Translation and Cross-Cultural Validation of Health Status Questionnaires. Eval. Health Prof..

[31] Reeve B.B., McFatrich M., Pinheiro L.C., Weaver M.S., Sung L., Withycombe J.S., Baker J.N., Mack J.W., Waldron M.K., Gibson D. (2017). Eliciting the child’s voice in adverse event reporting in oncology trials: Cognitive interview findings from the Pediatric Patient-Reported Outcomes version of the Common Terminology Criteria for Adverse Events initiative. Pediatr. Blood Cancer.

[32] Christodoulou C., Junghaenel D.U., DeWalt D.A., Rothrock N., Stone A.A. (2008). Cognitive interviewing in the evaluation of fatigue items: Results from the patient-reported outcomes measurement information system (PROMIS). Qual. Life Res..

[33] DeWalt D.A., Rothrock N., Yount S., Stone A.A., PROMIS Cooperative Group (2007). Evaluation of item candidates: The PROMIS qualitative item review. Med. Care.

[34] Irwin D.E., Varni J.W., Yeatts K., DeWalt D.A. (2009). Cognitive interviewing methodology in the development of a pediatric item bank: A patient reported outcomes measurement information system (PROMIS) study. Health Qual. Life Outcomes.

[35] Bond T., Yan Z., Heene M. (2020). Applying the Rasch Model: Fundamental Measurement in the Human Sciences.

